# Macroporous Poly(hydromethylsiloxane) Networks as Precursors to Hybrid Ceramics (Ceramers) for Deposition of Palladium Catalysts

**DOI:** 10.3390/molecules29163808

**Published:** 2024-08-11

**Authors:** Jan Mrówka, Robert Kosydar, Kamil Kornaus, Janusz Partyka, Magdalena Hasik

**Affiliations:** 1 Faculty of Materials Science and Ceramics, AGH University of Krakow, Al. Mickiewicza 30, 30-059 Krakow, Poland; jmrowka@agh.edu.pl (J.M.); kornaus@agh.edu.pl (K.K.); partyka@agh.edu.pl (J.P.); 2 Jerzy Haber Institute of Catalysis and Surface Chemistry, Polish Academy of Sciences, ul. Niezapominajek 8, 30-239 Krakow, Poland; robert.kosydar@ikifp.edu.pl

**Keywords:** polysiloxanes, ceramers, polyHIPEs, Pd catalysts, phenylacetylene hydrogenation

## Abstract

Poly(hydromethylsiloxane) (PHMS) was cross-linked with 1,3,5,7-tetramethyl-1,3,5,7-tetravinylcyclotetrasiloxane (D_4_^Vi^) in water-in-oil High Internal Phase Emulsions to form macroporous materials known as polyHIPEs. It was shown that in the process of pyrolysis under Ar atmosphere at 520 °C, the obtained polyHIPEs were converted to ceramers with high yields (82.8–88.0 wt.%). Structurally, the obtained ceramers were hybrid ceramics, i.e., they consisted of Si-O framework and preserved organic moieties. Macropores present in the polyHIPE precursors remained in ceramers. Ceramers contained also micro- and mesopores which resulted from the precursor’s mass loss during pyrolysis. Total pore volume and BET specific surface area related to the existence of micro- and mesopores in ceramers depended on the PHMS: D_4_^Vi^ ratio applied in polyHIPE synthesis. The highest total pore volume (0.143 cm^3^/g) and specific surface area (344 m^2^/g) were reached after pyrolysis of the precursor prepared with the lowest amount of D_4_^Vi^ as compared to PHMS. The composite materials obtained after deposition of PdO nanoparticles onto ceramers followed by reduction of PdO by H_2_ were active and selective catalysts for phenylacetylene hydrogenation to styrene.

## 1. Introduction

Polymer-derived ceramics (PDCs) are a group of ceramic materials obtained by thermal decomposition (pyrolysis) of macromolecular networks [1]. As compared to conventional techniques based on powder processing, such a method shows several advantages. It allows the preparation of ceramics at lower temperatures, and provides control over the structure, phase composition and thus properties of PDCs. Moreover, it utilizes good processability of macromolecular compounds: pyrolysis of polymers shaped into fibers or forming coatings on various substrates, after cross-linking, makes it possible to fabricate non-oxide ceramic fibers [2] or ceramic coatings [3] which otherwise are difficult to be obtained. There are also PDCs unattainable by conventional ways; silicon oxycarbides (SiOCs) are one of such systems.

SiOCs are analogues of amorphous silica, with some oxygen substituted for carbon atoms in the network [4]. Presence of carbon atoms results in good mechanical characteristics [5], high creep resistance [6] and good resistance to devitrification up to high temperatures [7]. Because of their unique properties, SiOCs-based ceramics are suitable for fabrication of coatings on TiAl alloy [8], stainless steel [9] or thermoelectric materials [10,11,12] protecting the substrates from high temperature corrosion. They can also be applied as biomedical [13,14], photoluminescent [15] materials as well as materials for anodes in lithium-ion batteries [16].

SiOCs-based ceramics are prepared by pyrolysis of polymeric precursors that contain both Si-C and Si-O bonds in their structure. Precursors can result from hydrolytic polycondensation of organoalkoxysilanes (the so-called sol-gel process) [8,9,10,11,12,14,15,16], hydrosilylation of multifunctional low-molecular-weight vinylsiloxanes [17,18] or cross-linking of polymeric siloxanes containing hydrocarbon (methyl, phenyl) as well as reactive (alkoxy, vinyl, Si-H) groups in their molecules [5,7,13,17]. Upon pyrolysis conducted in an inert (Ar) atmosphere, at temperatures between 700 and 1000 °C, precursors convert into ceramics which are frequently two-phase systems comprising SiOCs and graphitic-like carbon [17]. The precursor to ceramic transformation process usually involves several thermal decomposition steps, accompanied by evolution of gaseous products [19,20,21]. Final SiOC materials are, however, non-porous because the pores created due to the formation of gases sinter at higher temperatures. A range of methods have been proposed to prepare porous SiOCs-based ceramics. They include various techniques of direct foaming [22,23], addition of sacrificial fillers [24], replication of porous matrices [25] and HF etching of SiO_2_ domains formed at temperatures higher than 1000 °C [26].

It has been demonstrated that partial pyrolysis of SiOC precursors, conducted at 500–600 °C, leads to the materials of high specific surface area (up to ca. 600 m^2^/g) due to well-developed microporosity [27]. Such hybrid ceramics, with some organic groups still retained, are also called ceramers [28,29,30]. While micropores and—in case of some precursors [31] or upon addition of fillers [28]—mesopores are generated during pyrolysis, specific methods have to be applied to obtain ceramers additionally containing macropores. Thus, KCl as a water leachable template [31], emulsion templating [32], azodicarbonamide as a blowing agent [33] and expanded polystyrene beads as sacrificial template [34] were used. Macropores are critical for mass transport in the materials, whereas micro-/mesopores are important for adsorption/desorption processes. Ceramers derived from poly(methylsiloxane) (commercial MK resin) or poly(methylphenylsiloxane) (commercial H44 resin), with only microporosity developed, showed adsorption of hydrocarbons comparable to that of activated carbon, but with better desorption of the adsorbed species capability [28]. Partial pyrolysis of the precursors prepared using MK, H44 resins, (3-aminopropyl)triethoxysilane (APTES) and tetraethoxysilane resulted in various micro- and mesoporous hybrid ceramics that differed in surface characteristics [35]. They were suitable for the adsorption of either non-polar or polar solvents and CO_2_ [35].

High surface area makes ceramers perfect candidates for use as supports for heterogeneous catalysts. It was reported that hybrid ceramics with incorporated Pt particles catalyzed CO oxidation [29,30,34,36,37]. The ones containing Ni were active in CO_2_ methanation [38,39,40], while those with introduced Co were used in Fischer–Tropsch synthesis [38]. Precursors in these studies were obtained from H44 resin [37], H44 resin with the addition of APTES [29,30,34,37,38], MK or H44 resin mixed with bis[3-(trimethoxysilylpropyl]amine [39], MK resin and several amines [40] or by sol-gel method using APTES or APTES/phenyltriethoxysilane as reactants [36]. In the majority of cases, incorporation of metals into ceramers was an in situ process. Thus, these compounds were first mixed with an appropriate metal compound (hexachloroplatinic acid, nickel(II) acetate, nickel(II) acetylacetonate or nickel(II) nitrate hexahydrate, cobalt(II) nitrate), then cross-linked and finally pyrolyzed. To the best of our knowledge, there are only two publications [29,34] in which ceramer-metal systems were, for comparison, additionally prepared ex situ, i.e., by impregnation of a preformed ceramer with a sol of Pt nanoparticles. It is worth noting that ceramers with introduced metal particles investigated so far were microporous, i.e., contained exclusively pores generated upon pyrolysis [22,23,29,38], micro- and macroporous [30,34,40] or micro-, meso- and macroporous [39] when the precursors contained appropriate additives. It was shown for catalytic CO oxidation, however, that micropores restrict transport of reactants to the Pt centers in the catalyst [29]. Hence, for applications as catalytic carriers, ceramers with pores of various sizes are preferred.

The present work deals with micro-/meso-/macroporous ceramers prepared by partial pyrolysis of poly(hydromethylsiloxane) (PHMS)-based polyHIPEs, i.e., PHMS cross-linked with 1,3,5,7-tetramethyl-1,3,5,7-tetravinylcyclotetrasiloxane (D_4_^Vi^) in high internal phase water-in-oil emulsions. To illustrate their possible applications, the fabricated ceramers were impregnated with palladium(II) acetate (Pd(OAc)_2_) solution in toluene followed by heat treatment of the dried material in air to decompose the deposited Pd(OAc)_2_ to PdO. Finally, they were treated with H_2_ to reduce PdO to metallic Pd. The Pd-containing materials were tested as catalysts for phenylacetylene hydrogenation.

In this work, we demonstrate that a new type of precursors, i.e., polyHIPEs prepared from PHMS, can be applied for the preparation of hybrid ceramics (ceramers) containing macropores. Furthermore, we show that such ceramers can serve as supports for PdO particles. The composite materials obtained, after activation with hydrogen, are active and selective catalysts for hydrogenation of phenylacetylene to styrene. It should be noted that ceramer-Pd systems have not been described in the literature before. Additionally, there are no studies showing application of ceramer-metal systems as catalysts of phenylacetylene hydrogenation. Since this process is of industrial importance, our studies lead the way to new catalysts of potential wide use.

This work continues our earlier investigations on polysiloxane-derived polyHIPE-Pd materials [41,42,43].

## 2. Results and Discussion

### 2.1. Starting and Pyrolyzed Materials

In the studies, polyHIPEs obtained by cross-linking of PHMS with D_4_^Vi^ were pyrolyzed to obtain ceramers for use as supports for Pd catalysts (Section 3.2). As already mentioned, ceramers are well suited for such application since they show a high surface area attributed to high micro- and sometimes also mesoporosity. Care should be taken, however, when choosing pyrolysis temperature because porosity, and consequently surface area of PDCs, decreases as the temperature of the precursor’s pyrolysis increases [27]. Hence, if high porosity of the resultant material is required, the temperature of pyrolysis should be properly chosen.

Porosity of a ceramer is related to the amount of gaseous substances released upon thermal decomposition of the precursor at the temperature selected for the ceramer’s preparation. Types and quantities of volatiles evolved depend, in turn, on chemical structure as well as on cross-linking degree of the preceramic polymer. In our previous work, polyHIPEs were obtained from another polysiloxane, poly(methylvinylsiloxane), using different amounts of the cross-linker [42]. The resultant polyHIPEs showed different thermal properties [42]. In the present experiments, polyHIPEs were prepared at three molar ratios of Si-H groups from PHMS:Si-Vi groups from D_4_^Vi^ (Section 3.2.1) to verify if their pyrolysis would produce ceramers varying in porosity and surface area.

**Figure 1:**
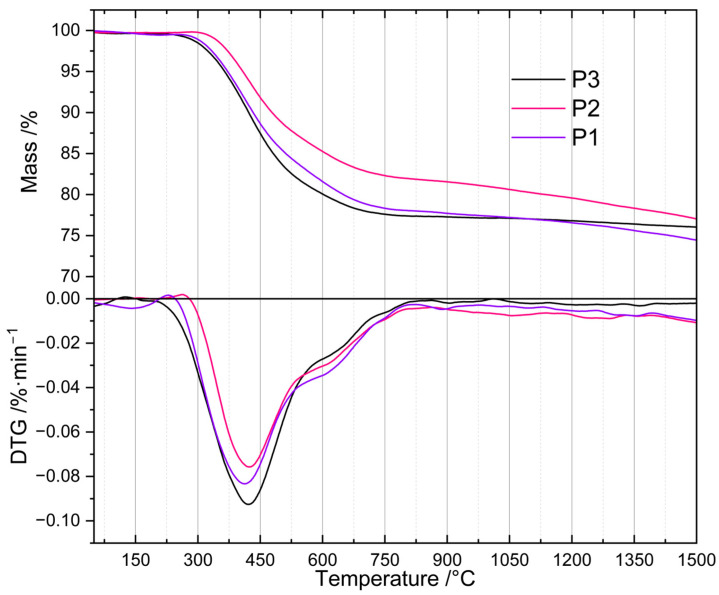
TG and DTG curves of the studied polyHIPEs. *Note:* for sample symbols please refer to Section 3.2.1.

To determine their thermal properties and to find the proper temperature for their pyrolysis, the prepared PHMS-based polyHIPEs were subjected to TG investigations (Section 3.3). TG curves (Figure 1) revealed that the materials differed in the onset temperature as well as in the rate of thermal decomposition. The P2 sample, obtained with the intermediate amount of D_4_^Vi^ as compared to PHMS (Si-H: Si-Vi groups molar ratio equal to 1:1, Section 3.2.1), started to degrade at ~320 °C. In the whole temperature range studied, its mass loss was slower than those of the P1 and P3 polyHIPEs prepared with the excess of Si-H and Si-Vi groups, respectively (Section 3.2.1). The latter materials, in turn, began to decompose at similar temperature of ~280 °C, but up to 1050 °C the P1 sample lost its mass more slowly than the P3 one. It should be noted that the mass of the P3 sample practically stabilized at ~750 °C indicating that its ceramization was complete at this temperature, whereas the masses of the P1 and P2 polyHIPEs decreased until the temperature of 1500 °C being the end of the measurements.

Differences in mass losses at temperatures in the range of 450–1000 °C were not large (Table 1). The highest one (5.2 wt.%) was observed between the P3 and P2 samples at 520 °C. At 1000 °C, the temperature often applied for the fabrication of SiOCs-based ceramics [16,22,23,24], mass losses found for the P1 and P3 materials were close (22.6 and 23.0 wt.%), while that of the P2 polyHIPE—owing to its slower thermal decomposition—was, respectively, by 2.7 wt.% and 3.1 wt.% lower. It is worth noting here that ceramic yields, around 80 wt.% in all cases, are high enough to consider the investigated polyHIPEs good precursors for SiOCs-based materials.

Above 1000 °C, the differences in mass loss were also low, being in the range between 1.0 and 2.6 wt.% at 1500 °C (Table 1). The materials generated at this temperature contained SiC whose formation was manifested by an exothermic peak at ~1300 °C visible in the DSC curves of the studied precursors. Transformation of SiOCs to amorphous SiO_2_ and crystalline SiC occurring above 1000 °C is well documented in the literature [44].

The observed differences in thermal properties of the investigated polyHIPEs can be explained by considering that during heat treatment, in addition to decomposition resulting in mass loss, ceramic precursors may undergo changes that lead to their further cross-linking. These are the reactions between Si-H and Si-CH_3_ or Si-CH_3_ and Si-CH_3_ groups of different polymer chains. They typically occur between 500 and 800 °C and generate Si-CH_2_-Si bridges between macromolecules and H_2_ or CH_4_ gases [4]. Additional cross-links make the systems more resistant to thermal decomposition which causes lower mass loss detected by TG. The slowest mass loss established for the P2 polyHIPE denotes that thermal decomposition processes were less significant, while cross-linking reactions were more pronounced in this system when compared to the remaining ones. Therefore, if PHMS/D_4_^Vi^ polyHIPEs were to be used as precursors to SiOC ceramics, the polymer should be cross-linked at the equimolar ratio of the reactive groups.

For the preparation of ceramers, however, the temperature of high rate of mass loss, connected with intensive release of volatiles, is of interest. As shown by derivative TG (DTG) curves (Figure 1), the investigated polyHIPEs lost their mass at a maximum rate at ~410 °C (P1 material), ~422 °C (P2 material) and ~425 °C (P3 material). We decided to perform pyrolysis at 520 °C (Section 3.2.2), i.e., above the temperature corresponding to the maximum decomposition rate for all the samples, but ensuring relatively large differences in their mass loss (Table 1). Mass drop of a material upon heat treatment depends mostly on the contribution of heavy gases in the decomposition products. Because of this, it is only a rough indicator of the amount of volatiles released at a given temperature. Nonetheless, we hoped that pyrolysis of the P1–P3 polyHIPEs conducted at 520 °C would enable us to produce ceramers characterized by different porosity and surface area.

Ceramers are hybrid inorganic/organic materials formed by incomplete transformation of polysiloxane precursors into SiOC ceramics. To determine their chemical structure, the initial polyHIPEs (P1–P3 samples) as well as the pyrolyzed materials (C1–C3 samples) were studied by FTIR spectroscopy using the ATR technique (Section 3.3). Additionally, thermal transformations of the P3 material were followed by measuring DRIFT spectra in situ upon its heating from room temperature up to 700 °C (Section 3.3).

**Figure 2:**
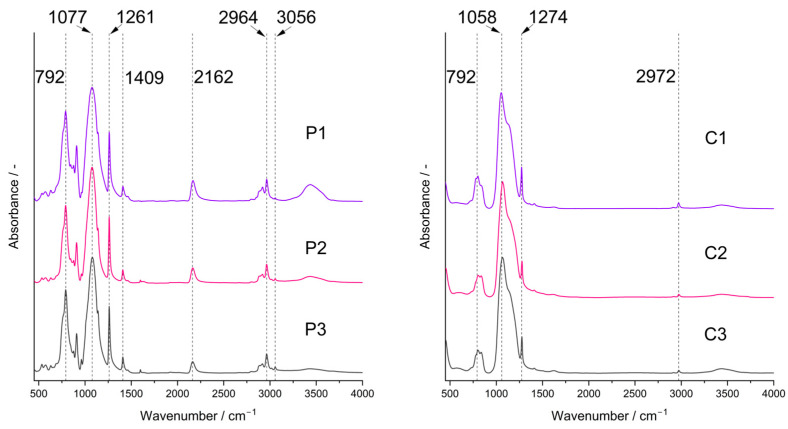
FTIR spectra of the studied polyHIPEs (P1–P3 samples) and ceramers (C1–C3 samples). *Note:* for sample symbols please refer to Section 3.2.1 and Section 3.2.2.

FTIR spectra confirmed the presence of bonds expected for PHMS cross-linked with D_4_^Vi^ in the initial systems and partial pyrolysis of the starting polyHIPEs occurring under the conditions applied in this work. In the spectra of the starting polyHIPEs (Figure 2, P1–P3 samples), there were bands ascribed to PHMS structure [45]: a strong one at 1077 cm^−1^ originating from the asymmetric stretching vibrations of Si-O-Si moieties in the polymer chains as well as the bands related to methyl substituents at 2964 cm^−1^ (C-H asymmetric stretching vibrations), 1409 cm^−1^ (C-H asymmetric bending vibrations) and 1261 cm^−1^ (C-H symmetric bending vibrations). Cross-linking of PHMS was evidenced by the bands at 2919 cm^−1^ and 2852 cm^1^ (C-H asymmetric and symmetric stretching vibrations, respectively) and a shoulder at 1142 cm^−1^ (C-H wagging vibrations), corresponding to the Si-CH_2_-CH_2_-Si bridges formed in the reaction between the polymer and D_4_^Vi^. Additionally, the bands due to reactive groups remaining in the cross-linked polymer could be seen, i.e., Si-H—at 2162 cm^−1^ (stretching vibrations) and vinyl groups—at 3056 cm^−1^ and 1598 cm^−1^ (C-H asymmetric stretching and C=C stretching vibrations, respectively). The band centered at ~3440 cm^−1^ proved in turn the formation of Si-OH groups via hydrolysis of some Si-H moieties during the preparation of polyHIPEs. It could be seen that intensities of the bands due to the Si-H and Si-OH groups were lower, whereas the bands originating from the vinyl groups were more distinct in the spectra of the polyHIPEs obtained with higher amounts of the cross-linker with respect to the polymer. This is understood since at a low amount of D_4_^Vi^, participation of Si-H groups in polymer cross-linking was limited. This favored their hydrolysis by water constituting the internal phase of the emulsion. At higher amounts of D_4_^Vi^ in the reaction medium, due to steric constraints, the involvement of all vinyl groups in polymer cross-linking was unlikely. Such a phenomenon was also observed in our earlier studies when poly(methylvinylsiloxane) was cross-linked using a cyclic hydrosiloxane, where at higher amounts of the cross-linker Si-H moieties remained [46]. The calculated ratios of integral intensities of the band due to Si-H groups at 2162 cm^−1^ and the band attributed to Si-CH_3_ groups at 1261 cm^−1^ (Section 3.3) were equal to 0.85, 0.78 and 0.56 in the spectra of the P1, P2 and P3 samples, respectively. Such values are in line with the increased content of the cross-linker in the systems.

FTIR spectra of the pyrolyzed materials (Figure 2, C1–C3 samples) showed a strong band corresponding to Si-O-Si vibrations located at 1058 cm^−1^. Shift of this band to lower wavenumbers with respect to the spectra of the initial polyHIPEs indicates a decrease in the average Si-O-Si bond angle taking place during pyrolysis as similar dependence was found for the spectra of silica glass [47]. On the other hand, for polysiloxane networks this band shifts to higher wavenumbers when the cross-linking degree in the system grows [48]. Hence, the change in the position of the Si-O-Si band in the spectra after pyrolysis may be due to disintegration of the network structure at higher temperatures. The spectra contained also the bands originating from the Si-CH_3_ groups at 2972 cm^−1^ and 1274 cm^−1^. However, they were of lower intensities than in the spectra of the corresponding P1–P3 polyHIPEs which corroborated thermal decomposition of a fraction of these groups. No bands due to Si-H or vinyl groups could be seen. Thus, studies by FTIR spectroscopy proved unequivocally that the C1–C3 materials were hybrid ceramics (ceramers), i.e., ceramics with some methyl groups still preserved. The materials contained also some Si-OH groups as shown by a broad band of a low intensity at ~3440 cm^−1^.

**Figure 3:**
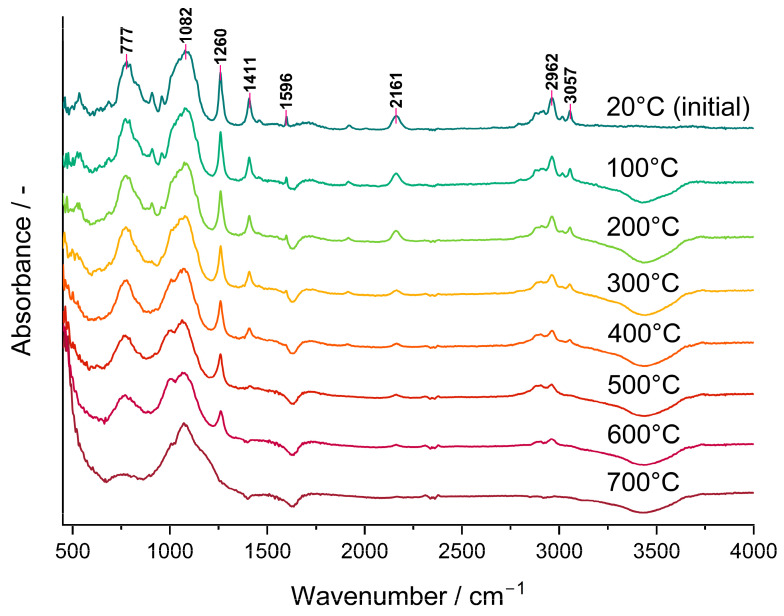
DRIFT spectra recorded in situ during heating of the P3 polyHIPE under Ar atmosphere.

The bands ascribed to Si-H as well as to CH_3_ groups lowered gradually in the FTIR spectra of the P3 polyHIPE measured in situ using the DRIFT technique (Figure 3). This demonstrated that their amounts in the system decreased as the temperature grew. It should be noted that a trace of the Si-H band at 2161 cm^−1^ was still visible in the spectrum of the material pyrolyzed at 600 °C in spite of its lack in that of the C3 ceramer (Figure 2) prepared from the P3 polyHIPE at 520 °C. This discrepancy may reflect the dynamics of the pyrolysis process during which various bonds (including Si-H) are formed and decomposed simultaneously. Alternatively, it may denote that the adsorbed/absorbed volatile Si-H group-containing species formed during pyrolysis did not evacuate from the sample before recording the spectrum. Nevertheless, pyrolysis conducted at 700 °C resulted in the complete loss of both Si-H and CH_3_ groups from the material which agrees with stabilization of its mass found in TG studies.

PolyHIPEs are macroporous materials whose partial pyrolysis was expected to result in the products with macropores retained, but containing also micro- and possibly mesopores. In view of the application of the prepared ceramers as catalyst carriers, investigations of their microstructure were of extreme importance. Macropores existing in the systems were examined by SEM while micro-/mesopores were examined by low temperature nitrogen adsorption studies (Section 3.3). Checking if macroporosity of the starting P1–P3 polyHIPEs changed upon pyrolysis was a special point of interest in the conducted SEM investigations.

**Figure 4:**
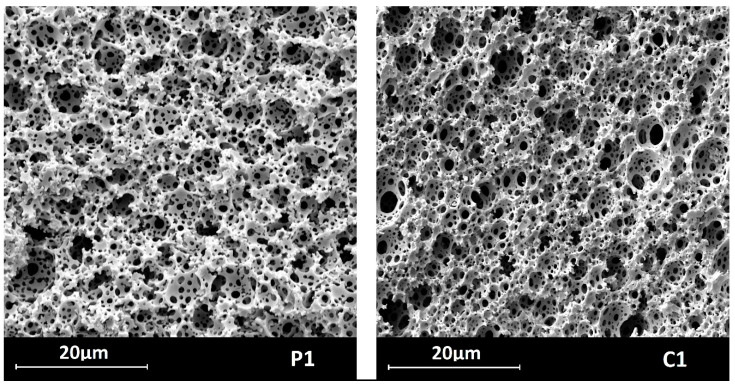
SEM images of the materials before (P1) and after (C1) pyrolysis.

SEM images (illustrated by those of the P1 and C1 samples in Figure 4, magnification: 5000×), showed that both P1–P3 and C1–C3 materials—like typical polyHIPEs [49,50]—contained two types of macropores: larger ones, often called voids, and smaller ones by which voids were interconnected called windows. Hence, the materials exhibited open macroporosity; qualitatively, pyrolysis did not affect the microstructure of the samples.

**Figure 5:**
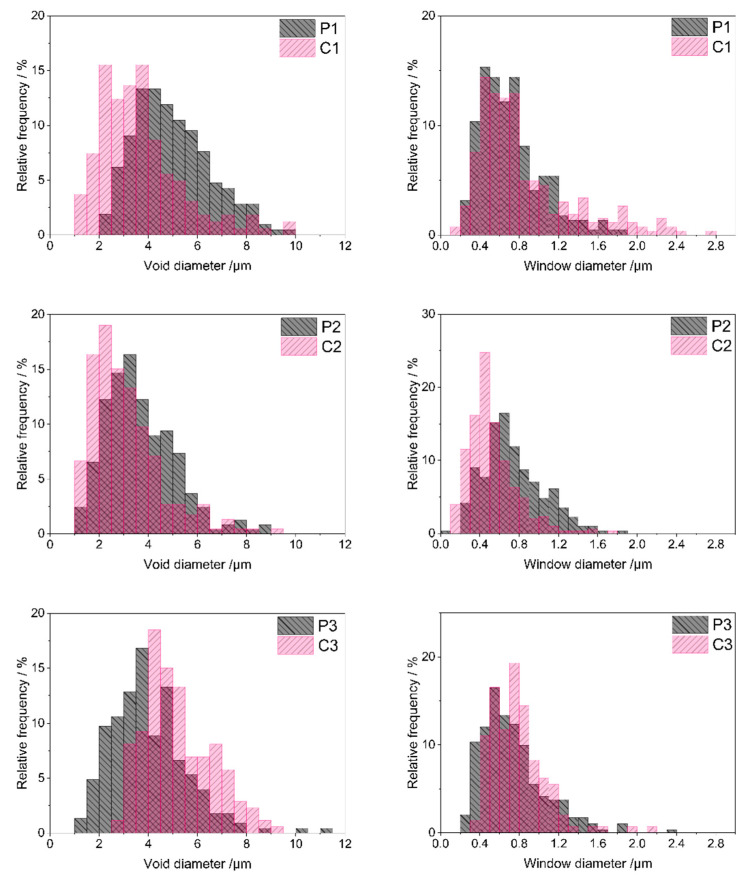
Void and window size distributions in the studied polyHIPEs and ceramers determined by analysis of SEM images. *Note:* for sample symbols please refer to Section 3.2.1 and Section 3.2.2.

Quantitative analysis of SEM images performed using ImageJ 1.53k software (Section 3.3) made it possible to determine sizes as well as size distributions of voids and windows in the materials. As can be seen in the size distribution diagrams (Figure 5), most voids in the starting polyHIPEs showed diameters in the range of 2–9 μm (P1 material), 1–6.5 μm (P2 material) and 1–8 μm (P3 material). Low shares of larger voids were present in the systems as well. The maximum void diameter of 11.2 μm was found for the P3 polyHIPE, whereas diameters of voids existing in the P1 and P2 polyHIPEs did not exceed 10 μm (Table 2). Presence of larger voids resulted in relatively high mean and median diameter values for the P1 and P3 polyHIPEs when compared with the P2 one (Table 2). It is also worth noting that the P1 material contained the lowest fraction of the smallest voids, of a diameter below 4 μm (Table 2).

Voids replicate sizes and distribution of internal phase droplets in the emulsion during preparation of polyHIPEs and are connected with the stability of the emulsion in the time of the process [50]. PolyHIPE formation involves creation of a polymer network around internal phase droplets. It must be fast enough to occur before the inherently unstable high internal phase emulsion destabilizes. The presence of large voids together with the lowest fraction of the smallest, and hence, the highest fraction of larger voids observed in the P1 material, imply the slowest network generation in this system. This can be explained by slow polymer cross-linking resulting from the lowest amount of the cross-linker with respect to the polymer in the P1 emulsion (Section 3.2.1). Cross-linking of the polymer in the P2 and P3 systems, containing higher amounts of D_4_^Vi^ (Section 3.2.1), was faster. This led to higher shares of small voids in the P2 and P3 materials as compared with the P1 one (Table 2).

Pyrolysis evidently led to sintering of some voids in the P1 and P2 polyHIPEs. Minimum void diameters in the C1 and C2 ceramers were lower and fractions of smaller voids higher than in the respective initial polyHIPEs (Figure 5, Table 2). Degree of sintering—expressed as the increase in the share of voids with diameter below 4 μm—was higher in the case of the P1 polyHIPE: the C1 ceramer contained 2.2 times more small voids than its precursor, while pyrolysis of the P2 material resulted in the 1.2-fold increase in the fraction of small voids (Table 2). In contrast, upon thermal transformation of the P3 polyHIPE to the C3 ceramer, the minimum void diameter (Table 2) and the share of larger voids grew markedly (Figure 5). Consequently, the fraction of voids with a diameter lower than 4 μm in the C3 ceramer was ca. 3 times lower than in the P3 polyHIPE (Table 2). Because of such changes, the mean and median diameters of voids in the C1, C2 ceramers were lower and in the C3 ceramer higher than in the respective initial polyHIPEs (Table 2).

Altered void sizes in ceramers show that low-temperature pyrolysis influences macropores existing in the precursors. Since pyrolysis performed at higher temperatures causes loss of transient porosity [27], sintering of some voids found for the P1 and P2 polyHIPEs is not surprising. Expansion of voids in the P3 material and its different behavior from the other precursors are, however, quite unexpected. The exact reasons for these phenomena are not known at the moment. They may be related to the differences in mass losses of the precursors upon pyrolysis. For the P3 one, the highest mass loss at 520 °C was detected in TG investigations (Table 1) indicating the highest amount of heavy gases evolved during its thermal decomposition. Mass loss of the P2 material was the lowest (Table 1) which implies the lowest amount of heavy volatiles formed. As suggested in Ref. [51], diameters of pores generated upon thermal transformations of preceramics depend on the molecular volumes of pyrolysis products. It seems therefore possible that heavy gaseous pyrolysis products, of larger molecules, caused expansion of voids during pyrolysis of the P3 polyHIPE. Additionally, it can be noticed that the highest degree of void sintering was observed for the P1 polyHIPE, containing the lowest fraction of the smallest voids among the studied precursors (Table 2). Thus, pyrolysis has a strong effect on large voids present in the initial polyHIPEs.

Diameters of most windows existing in the starting polyHIPEs (Figure 5) were in the range of 0.2–1.4 μm (P1 sample) or 0.2–1.6 μm (P2 and P3 samples). Low contributions of smaller or larger windows caused some differences in minimum or maximum window diameter values found for the systems (Table 2). The calculated mean and median window diameters were, however, very close for all the polyHIPEs (Table 2). This means that in all starting materials, pores that connected voids were quite similar.

According to some researchers, windows in polyHIPEs are formed due to the contraction of the emulsion oil phase volume at the polymer gel point [52]. Others claim that windows are related to post-synthesis treatment of the materials [53]. Similarity of window sizes in the studied PHMS-based polyHIPEs seems to support the second theory as all the materials were treated in the same way after synthesis (Section 3.2.1). If the first theory was valid, window sizes should have been different in the systems obtained with various amounts of D_4_^Vi^ due to various cross-link densities.

Pyrolysis resulted in significant sintering of some windows in the P2 material: C2 ceramer contained more small windows than the starting polyHIPE (Figure 5). This was manifested by an almost two-fold increase in the share of windows with a diameter below 0.6 μm in the material after pyrolysis (Table 2). Moreover, minimum (if a low share of windows with a diameter below 0.1 µm, seen in the size distribution diagram of the P2 material in Figure 5, is neglected), maximum, mean and median window diameters lowered during pyrolysis of the P2 polyHIPE (Table 2). The opposite was observed in the case of the P1 and P3 precursors: lowering in the fractions of small windows and an increase in the maximum window diameter were the main outcomes of their pyrolysis (Figure 5, Table 2). The effect was more pronounced in the P3 polyHIPE. Thus, thermal decomposition of the studied polyHIPEs influenced not only voids, but also windows, i.e., smaller macropores existing in the precursors. Interestingly, pyrolysis led to sintering of both voids and windows in the P2 material, expansion of voids and windows in the P3 polyHIPE, while in the P1 one, voids sintered and windows expanded (Table 2).

**Figure 6:**
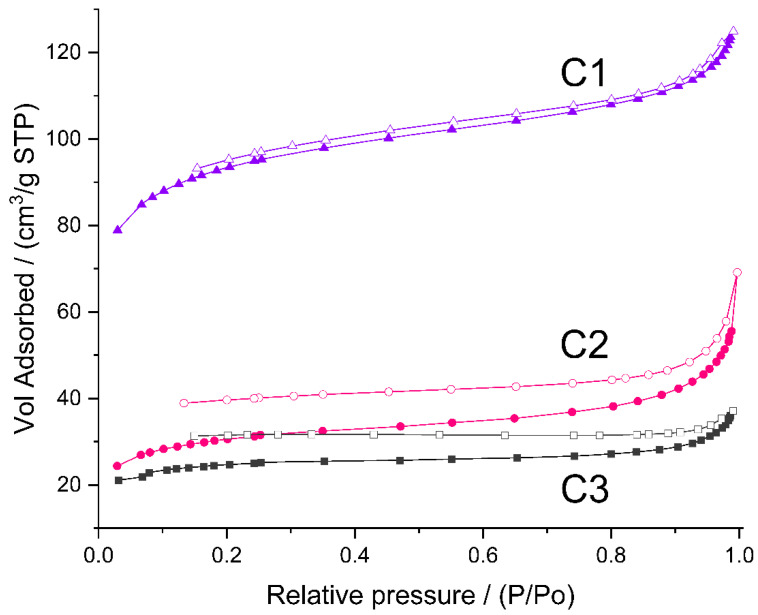
N_2_ adsorption/desorption isotherms of the studied ceramers. *Note:* for sample symbols please refer to Section 3.2.2.

As mentioned earlier, to study micro-/mesoporosity of the prepared materials, low-temperature nitrogen adsorption experiments were carried out. Nitrogen did not adsorb on the initial P1–P3 polyHIPEs. The adsorption isotherms of C1–C3 ceramers (Figure 6) can be considered as type II according to IUPAC classification, characteristic for macroporous or non-porous solids [54,55]. However, high adsorption at low relative pressure (p/p_0_) found for all the samples pointsto the presence of micropores. Hysteresis between adsorption and desorption branches of the isotherms implies in turn the existence of mesopores in the ceramers. The hysteresis loop due to capillary gas condensation in mesopores usually closes at p/p_0_ ≈ 0.4 [ 54,55]. In the case of the studied ceramers, hysteresis extended into a low p/p_0_ range indicating that micropores present in the materials were of about the same sizes as the adsorptive molecules [55]. All measured hysteresis loops were open which showed that in the course of measurements, the adsorbate was not removed completely upon the lowering of pressure. Open hysteresis loops at low relative pressures were recorded also for the N_2_ adsorption/desorption isotherms of other ceramers [27]. This phenomenon was, however, not discussed in this paper.

The volume of N_2_ adsorbed (Figure 6) showed that among the studied ceramers, C1 was characterized by the highest, while C3 was characterized by the lowest porosity. Calculations performed in the way recommended in Ref. [56] (Section 3.3) demonstrated that total pore volume in the C1 sample (0.143 cm^3^/g) was ca. 3.5 and 4 times higher than those in the C2 (0.041 cm^3^/g) and C3 (0.036 cm^3^/g) ones, respectively (Table 3). Micropores were the main fraction of pores detected by N_2_ adsorption in all the studied materials: ratios of micropore to mesopore volume ranged from 1.2 for C2 to 2.6 for C3 ceramer (Table 3). The highest volumes of both micro- and mesopores contained the C1 sample (Table 3). Differences in their porosity were manifested in the specific surface area related to micropores (S_BET_) and the remaining surface (S_ext_) of the materials. They were the highest in the case of the C1 ceramer (Table 3).

The highest porosity of the C1 ceramer must be related to the highest fraction of low-molecular-mass gases released upon pyrolysis of its precursor, P1 polyHIPE, as compared to P2 and P3 materials. This becomes especially clear when one takes into account that at the temperature of ceramers’ preparation (520 °C), the difference in mass losses of P1 and P3 materials found in TG investigations (Table 1) was not as significant as the difference in porosity and surface area of C1 and C3 ceramers (Table 3). It should be reminded here that P1 polyHIPE was prepared with the lowest amount of the cross-linker with respect to the polymer (Section 3.2.1) and, as confirmed by FTIR spectroscopy, concentration of Si-H groups in this sample was the highest. These groups can participate in a number of thermal reactions that lead to the formation of low-molecular-mass compounds [19]. In such cases, high amounts of gases evolved are accompanied by low mass loss which explains the high porosity of the C1 ceramer in spite of the relatively low mass drop of its precursor during pyrolysis.

Thus, our studies demonstrate unambiguously that the cross-linker/polymer ratio applied in the preparation of PHMS-based polyHIPEs controls the porosity and surface area of ceramers obtained by their partial pyrolysis.

### 2.2. Ceramers with Introduced Palladium

To prepare palladium-containing materials, the C1–C3 ceramers were impregnated with Pd(OAc)_2_ solution in toluene, and then—after drying at room temperature—heated in air at 350 °C to decompose the deposited Pd(OAc)_2_ to PdO (Section 3.2.2). The C1_Pd–C3_Pd materials thus prepared were subjected to H_2_ treatment before performing catalytic tests with the aim of reducing PdO to metallic Pd (Section 3.4).

The samples with introduced PdO were characterized by XRD and TPR (Section 3.3). Catalytic properties of the materials treated with H_2_ were investigated in phenylacetylene hydrogenation (Section 3.4).

**Figure 7:**
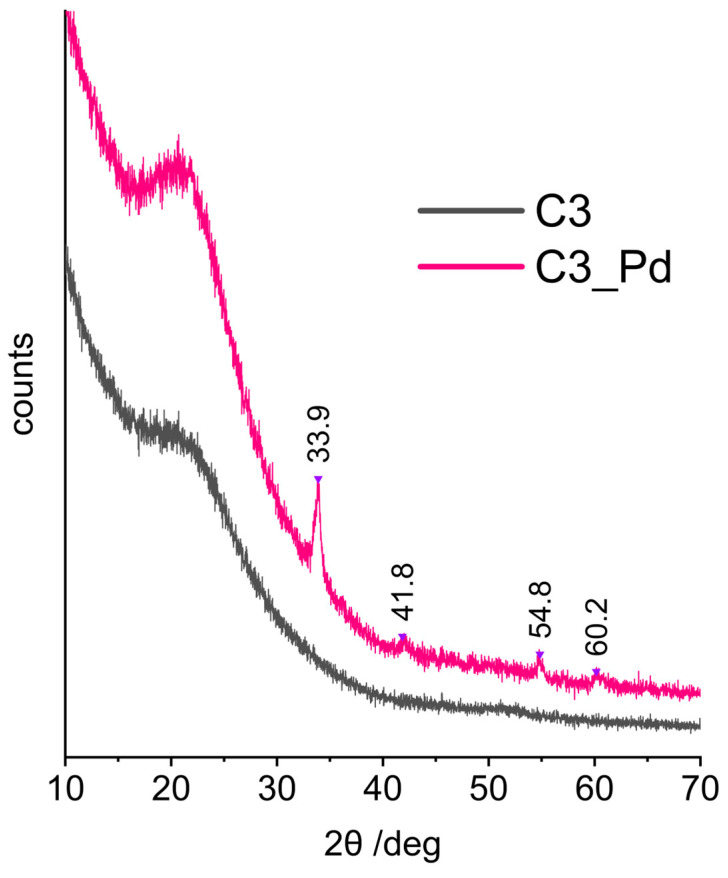
XRD diffraction patterns of the C3 ceramer and the C3_Pd material. *Note:* for sample symbols please refer to Section 3.2.2.

XRD patterns (represented in Figure 7 by that of C3_Pd material) confirmed that C1_Pd-C3_Pd systems contained PdO. This was evidenced by the reflections at 2θ angle values equal to 33.9°, 42.0°, 54.8° and 60.2° corresponding to, respectively, (101), (110), (112) and (103) planes in a tetragonal PdO crystal lattice [57]. It should be noted that there were no signs of Pd(OAc)_2_ presence whose most intensive reflection in the X-ray diffractogram should be at 2θ = 11° [58]. This proved that in the adopted experimental conditions Pd(OAc)_2_, initially deposited on ceramers, was completely transformed to PdO.

Calculations performed using the Scherrer equation based on the (101) reflection showed that the average sizes of PdO crystallites were equal to 15.6, 14.2 and 17.3 nm for C1_Pd, C2-Pd and C3_Pd materials, respectively. These values, when related to pores, are in the mesopore range. Hence, assuming that PdO detected by XRD was deposited in the pores of a ceramer, it can be concluded that C3 contained the largest while C2 contained the smallest mesopores. The same relationship was true for the macropores (both voids and windows) present in the materials: C3 contained the highest fractions of large macropores and C2 contained the highest fraction of small macropores (Table 2).

Moreover, from the XRD studies, it followed that the materials with incorporated PdO were composites. The broad reflection centered at 2θ angle of ca. 20°, seen in the XRD patterns of the starting ceramers (Figure 7, C3 sample), was preserved in those of the PdO-containing systems (Figure 7, C3_Pd sample). Thus, PdO was dispersed in amorphous ceramer matrices. The reflection at 2θ = ~20–22° was observed also in the XRD patterns of other amorphous network structures containing Si and O atoms, such as silica [59] or cross-linked polysiloxanes [60,61,62].

**Figure 8:**
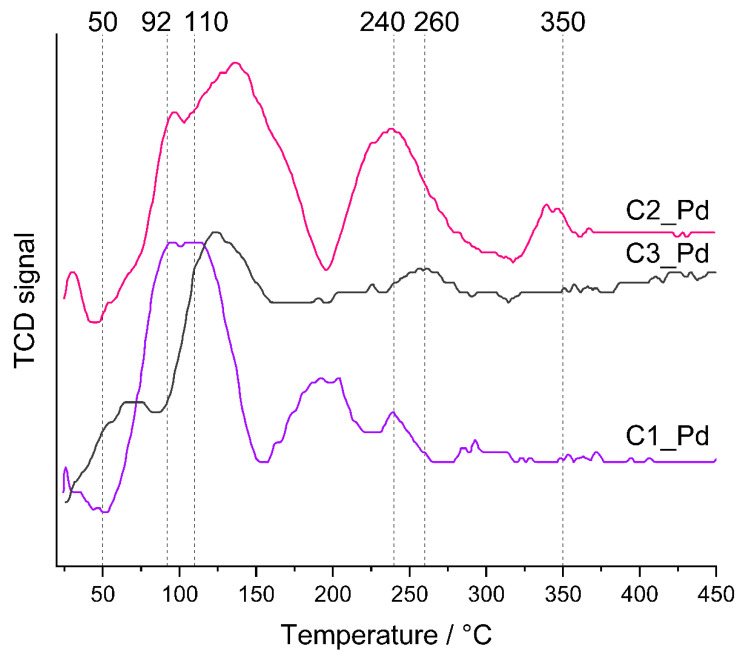
TPR profiles of the prepared ceramer-PdO systems. *Note:* for sample symbols please refer to Section 3.2.2.

TPR profiles (Figure 8) showed that the reducibility of PdO particles present in C1_Pd-C3_Pd materials was different. In all cases, hydrogen consumption—represented by maxima in the TPR curves—was a multistep process. It lasted to ~350 °C for the C2_Pd material, whereas for the C1_Pd and C3_Pd ones it was finished at ~240 °C and ~260 °C, respectively. It should be mentioned here that bulk PdO is readily reduced to metallic Pd at subambient temperatures [63]. Reduction of PdO deposited within another material may occur at higher temperatures, whose values depend on the interactions between oxide particles and the matrix, shapes and sizes of oxide particles as well as on their dispersion in the material. In particular, a decrease in particle size shifts the reduction temperature to higher values: small PdO particles deposited on alumina exhibited reduction maxima at temperature as high as 320 °C [64]. Other researchers, however, assigned the high temperature maxima (355 °C, 363 °C) occurring in the TPR profiles of Pd/Al_2_O_3_ catalysts to reduction of both small PdO particles and hydroxyl groups of the support [65]. Such assignment is also possible for our ceramers where—according to FTIR spectra (Figure 2)—some hydroxyl groups remained.

Lower temperature TPR maxima, unequivocally related to PdO reduction, revealed that the C1_Pd material contained the most easily reduced PdO particles. The first maximum in its TPR profile appeared at ~25 °C; the next, composed of two maxima (possibly due to PdO particles differing in the strength of interactions with the support), at ~92 °C and ~110 °C. Reduction of PdO present in the C2_Pd sample was more and in the C3_Pd one was the most difficult. Their TPR curves showed the maxima at higher temperatures: ~30 °C, ~95 °C and ~130 °C (C2_Pd material) and at ~125 °C (C3_Pd material).

Additionally, TPR profiles of the C1_Pd and C2_Pd materials contained the negative peak at ~45–50 °C that could be attributed to the decomposition of Pd hydrides. No such signal was seen in the TPR curve of the C3_Pd sample. It is known that absorption of hydrogen by Pd particles larger than 2 nm results in Pd hydrides which are easily decomposed at higher temperatures giving rise to negative peaks in TPR patterns [63,64,65]. Absence of the negative peak in the TPR curve of the C3_Pd material suggests then that it contained the smallest, well-dispersed Pd particles.

For catalytic investigations, the process of phenylacetylene hydrogenation was selected because it allows the testing of properties of redox catalysts under mild conditions [66]. Moreover, this reaction is industrially important as it is applied to remove phenylacetylene contaminant from styrene monomer before polymerization [67]. Phenylacetylene hydrogenation proceeds in two consecutive steps: the first one yields styrene which is then further hydrogenated to ethylbenzene. In view of the industrial application of the process, active catalysts that ensure high selectivity to styrene are desired.

**Figure 9:**
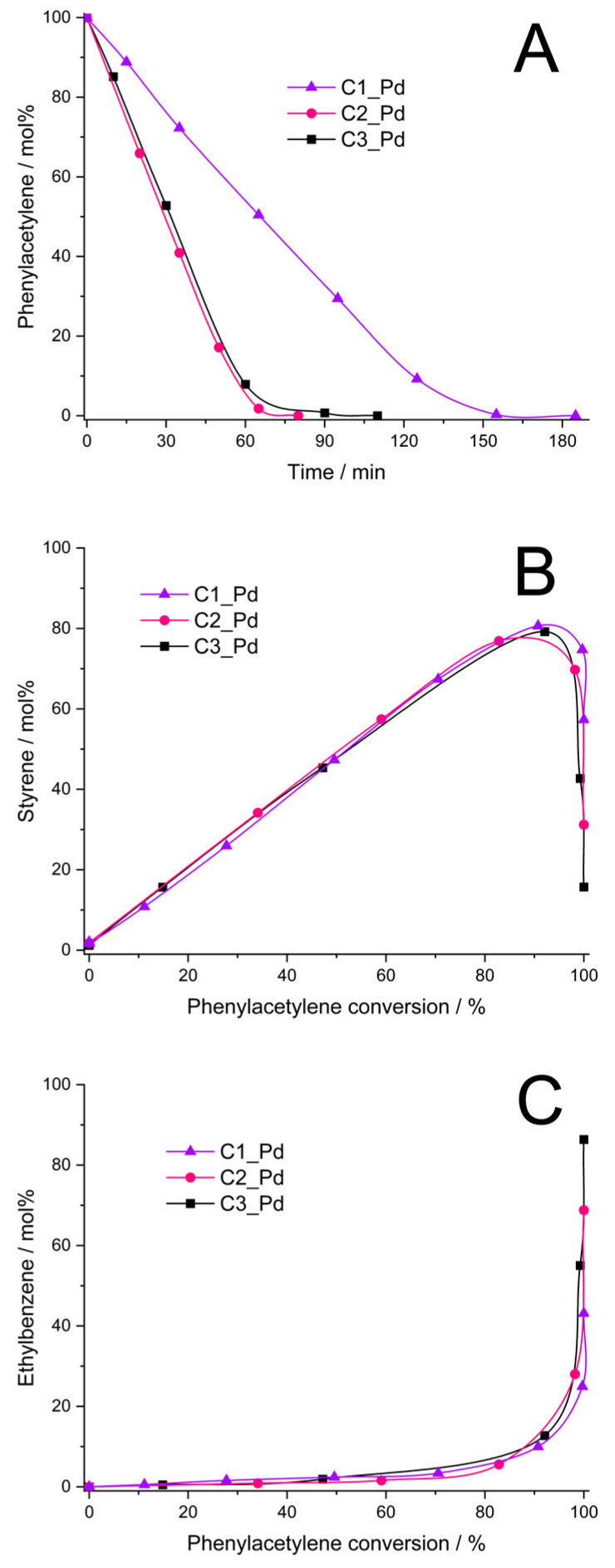
Results of phenylacetylene hydrogenation catalyzed by C1_Pd, C2_Pd and C3_Pd materials treated with H_2_ (**A**–**C**). *Note:* for sample symbols please refer to Section 3.2.2.

It was found that the C1_Pd–C3_Pd materials, after reduction with H_2_ (Section 3.4), were catalytically active in phenylacetylene hydrogenation. The catalysts obtained from C2_Pd and C3_Pd precursors showed comparable activity (initial rates of the hydrogenation process equal to 0.16 and 0.14, respectively, Figure 9A, Table 4), while the activity of the catalyst obtained from the C1_Pd sample was 2.3–2.7 times lower (initial rate of hydrogenation: 0.06 mol/min⋅g Pd, Figure 9A, Table 4). Styrene yield was close for all the catalysts, but it reached its maximum at a different phenylacetylene conversion (Figure 9B, Table 4). Thus, the maximum styrene yield of ~76% was attained at ~85% phenylacetylene conversion for the C2_Pd-derived sample, 78% at 90% conversion for the C3_Pd-based material and 81% at 92% conversion for the one originating from the C1_Pd precursor. Then, the yield to styrene decreased quickly for each sample. This was accompanied by the abrupt growth of ethylbenzene yield (Figure 9C). Maximum selectivity to styrene, S_max_, was equal to 89.4%, 86.7% and 88.0% for the C2_Pd-, C3_Pd- and C1_Pd-derived materials, respectively (Table 4). As reported in the literature, selectivity to styrene of phenylacetylene hydrogenation on other catalysts ranged from 60% at 99% phenylacetylene conversion (catalyst: Pd/Al_2_O_3_, [68]), through 86–90% (catalyst: Pd/TiO_2_, [69]) and 88.5–90.5% (catalysts: powdered Pd_2_Ga, PdGa, elemental Pd, [70]) at 100% phenylacetylene conversion up to 96% at 99% phenylacetylene conversion (catalyst: Pd/TiO_2_, [71]). Although comparison should be taken with care since the catalytic process by various research groups was performed in different conditions, it demonstrates a great potential of the ceramer-supported Pd catalysts studied in the present work. It should be pointed out that out our catalytic investigations were aimed at illustrating the possible applications of the prepared new materials. Because of this, in this work, we did not optimize parameters of phenylacetylene hydrogenation.

As found in the studies, ceramers used for deposition of PdO varied in porosity (Table 3). According to XRD, PdO introduced into ceramer matrices was of various average crystallite sizes and, as revealed by TPR, PdO particles of different reducibility existed in the systems. All these factors could have influenced the catalytic properties of the materials. Out of these parameters, porosity seems to be of primary importance. This is because reduced C2_Pd and C3_Pd materials, prepared using ceramers of close porosity, showed similar catalytic activity, while average PdO crystallite sizes present in these materials and their reducibility were different. Reduced C1_Pd catalyst was the least active, even though it contained PdO crystallites of intermediate average size in comparison with the other ones; they were of relatively good reducibility. The C1 matrix, applied for fabrication of C1_Pd material, showed significantly higher porosity than the other ones. To establish the mechanism deciding on a different catalytic performance of the studied materials, more investigations would be needed. Most probably, however, high porosity of the C1 support limited diffusion of the reactants to Pd centers located in catalyst’s micropores and/or made some of the catalytic centers inaccessible for the reactants. High selectivity to styrene indicates in turn that its adsorption on ceramer-supported Pd centers was weak which prevented it from further hydrogenation to ethylbenzene. This was reported to occur on electron-rich Pd catalytic sites [72]. On the other hand, in Ref. [73] it was proposed that high selectivity to styrene is ensured when associative adsorption (involving C≡C π bonds) of phenylacetylene on the Pd surface takes place. This could be the case in our systems.

Thus, our studies show that for use as supports for catalysts, ceramers obtained from PHMS-based polyHIPEs prepared with higher amounts of cross-linker are preferred.

## 3. Experimental Section

### 3.1. Materials

PHMS, D_4_^Vi^, dimethylsiloxane-25–30% ethylene oxide copolymer (DBE-224) and Karstedt catalyst solution in xylene (~2 wt.% of Pt) were supplied by ABCR (Karlsruhe, Germany). Palladium(II) acetate (≥99.9%, trace metals basis) was purchased from Merck (Poznań, Poland). Chlorobenzene, toluene, tetrahydrofuran and acetone were supplied by Avantor Performance Materials S.A. (Gliwice, Poland). Chlorobenzene was dried over anhydrous potassium carbonate and distilled from P_4_O_10_. Toluene and tetrahydrofuran were dried over potassium hydroxide pellets and distilled from sodium/benzophenone in the atmosphere of argon. All other chemicals were applied without any preliminary treatment.

### 3.2. Preparation Methods

#### 3.2.1. Formation of Preceramic Foams

Siloxane-based polyHIPEs were prepared by cross-linking of PHMS with D_4_^Vi^ in water-in-oil high internal phase emulsions (HIPEs) using the procedure similar to that described in Ref. [41]. Briefly, the oil phase was formed by mixing the polymer, the cross-linking agent, DBE-224 and chlorobenzene in a glass vial. Then, Karstedt catalyst was added under constant stirring. The oil phase was immediately transferred into an agate mortar and mixed carefully using a pestle with the aqueous phase (0.02 M NaCl solution in water) added dropwise to constitute 82% of the emulsion by mass. After the complete addition of the aqueous phase, viscous white emulsion was transferred into a PTFE crucible and heated at 80 °C for 24 h. The obtained monoliths were cut into small cubic blocks (around 4 mm) using a razorblade and washed with acetone in a Soxhlet apparatus for 24 h. Finally, the obtained materials were dried in air at room temperature and washed in dry tetrahydrofuran. After this, the samples were dried at room temperature.

PolyHIPEs were prepared at three molar ratios of reactive groups, Si-H (from PHMS): Si-Vi (from D_4_^Vi^) equal to 1:0.66, 1:1 and 1:1.5. In a typical experiment, the oil phase of HIPE contained 1 g of PHMS, 0.94/1.43/2.14 g of D_4_^Vi^, 0.49/0.61/0.79 g of DBE-224 and 0.61/0.76/0.98 g of chlorobenzene (depending on the molar ratio of reactive groups); 7 μL of Karstedt catalyst solution were added to it.

In this work, symbols P1, P2 and P3 will be used to denote polyHIPEs obtained at Si-H:Si-Vi groups molar ratio equal to 1:0.66, 1:1 and 1:1.5, respectively.

#### 3.2.2. Pyrolysis and Impregnation with Palladium

The monolithic blocks were subjected to pyrolysis at 520 °C in Ar atmosphere in the quartz tube furnace. Around 500 mg of the material on a graphite mat were placed in the furnace. Argon flow was maintained for 30 min to ensure full air evacuation and then the heating rate of 5 °C/min was applied. The material was kept at 520 °C for 2 h followed by free cooling to room temperature. The obtained off-white ceramers were crushed in agate mortar and solution of Pd(OAc)_2_ in toluene (2 mg of Pd per 1 cm^3^ of toluene) was added dropwise with constant stirring to fill the material’s pores completely. The amount of Pd(OAc)_2_ used was calculated to obtain 1 wt.% Pd/ceramer composites. After drying in air, the materials were placed in quartz crucibles and kept in a chamber furnace, in air at 350 °C (ramp rate: 10 °C/min) for 2 h to fully decompose Pd(OAc)_2_ to PdO.

Further on in this paper, the symbols C1, C2, and C3 are assigned to ceramers obtained from P1, P2, and P3 polyHIPEs, respectively, while the corresponding ceramers with deposited PdO are referred to as C1_Pd, C2_Pd, and C3_Pd.

### 3.3. Characterization Methods

FTIR spectra of initial and pyrolyzed samples were collected using ATR (attenuated total reflectance) method in the range of 550–4000 cm^−1^ on a BIO-RAD FTS6000 (Bio-Rad, Hercules, CA, USA) spectrometer. It was equipped with ZnSe crystal; 45° incident beam angle was used. For the P3 sample, the diffused reflectance IR (DRIFT) spectra in the 400–4000 cm^−1^ range were additionally recorded in situ_._ The material was heated in the spectrometer in Ar atmosphere from room temperature to 700 °C at the rate of 10 °C/min. Resolution of all IR measurements performed in this work was equal to 4 cm^−1^. A total of 64 scans were collected for each ATR and 128 scans for each DRIFT spectrum. Based on ATR FTIR spectra of the starting polyHIPEs, ratios of integral intensities of the band due to Si-H groups at 2162 cm^−1^ and that originating from Si-CH_3_ groups at 1262 cm^−1^ were calculated.

Thermogravimetric (TG) studies were performed on an NETSCH STA 449F3 (Netzsch Gerätebau GmbH, Selb, Germany) apparatus. The sample (ca. 10 mg) was placed in Al_2_O_3_ crucible and heated to 1500 °C with a heating rate of 5 °C/min under Ar flow.

SEM micrographs were taken by Phenom XL scanning electron microscope (Thermo Fisher Scientific, Eindhoven, Netherlands) using the secondary electron detector (SED). Samples of around 4 × 4 × 2 mm were mounted on Al stabs using nickel-based paste and coated with 40 nm of gold prior to analysis. Based on SEM images, diameters of pores present in the materials were determined in the way described in Ref. [43]. In SEM image analyses, the ImageJ 1.53k software was applied and 220–300 pores were measured.

N_2_ adsorption measurements were performed on ASAP 2010 unit (Micromeritics Instrument Corporation, Norcross, GA, USA) using 99.999% N_2_ (Air Liquide, Krakow, Poland). Before the tests, samples were degassed at 200 °C for 24 h to remove all impurities. The obtained adsorption data were analyzed as recommended for microporous materials in Ref. [ 56. Thus, to calculate specific surface area of micropores (S_BET_), the BET equation was applied in the relative pressure (p/p^0^) range where the BET plot was a straight line whose intercept with y-axis (called BET constant, denoted as C) was positive and, additionally, the term n(p^0^-p) (where n—amount of the adsorbed nitrogen) increased continuously as the p/p^0^ grew. Such p/p^0^ range established for the ceramers studied in this work was between 0.03 and 0.12 and therefore S_BET_ was calculated in this range. The external surface area, micropore volume and total pore volume of the ceramers were determined using the *t*-plot method in which the Harkins–Jura isotherm served as the reference. The micropore volume was evaluated as the intercept of the extrapolated linear fit of the modified isotherm in the low-pressure range (p/p^0^ = 0.08–0.20) with the y-axis, the external surface area (S_EXT_)—as the slope and the total pore volume—as the intercept with the y-axis of the extrapolated linear fit of the modified isotherm in the high-pressure range (p/p^0^ = 0.25–0.95). Mesopore volume was calculated by subtracting the micropore volume from the total pore volume.

Temperature-programmed reduction (TPR) was carried out using a Chembet-3000 (Quantochrome Instruments, Boynton Beach, FL, USA) apparatus equipped with a thermal conductivity detector (TCD). In the measurements, ca. 0.015 g of a sample was placed in a quartz reactor and heated in the flow of 5 vol.% H_2_ in Ar (flow rate: 30 mL/min) at the rate of 10 °C/min from room temperature to 500 °C.

X-ray diffractometry (XRD) data were collected by PANalytical Empyrean powder diffractometer (Malvern Panalytical, Almelo, The Netherlands) using Kα radiation from Cu anode. The transmission mode configuration with rotating sample was used. The primary beam setup consisted of focusing mirror and 1/32° molybdenum divergence slit. All measurements were carried out at room temperature and under ambient pressure.

### 3.4. Catalytic Tests

Catalytic hydrogenation of phenylacetylene (PhAc) was performed in an agitated batch glass reactor under atmospheric pressure of hydrogen at room temperature using the same procedure as that applied in our previous work [41,43]. An amount of 20 mg of Pd-containing sample was placed in a flask followed by the addition of 20 mL of THF. The mixture was sonicated for 15 min to ensure good dispersion of the material in the solvent. It was then transferred to the glass reactor, which was affixed to the platform shaker, and another 20 mL of THF was added. N_2_ was flushed through the reactor for 20 min followed by H_2_ flow for 30 min to reduce PdO to metallic Pd. After this time, 200 µL of PhAc were introduced by syringe and the hydrogenation reaction began. Shaking of the reactor was carried out at such a speed to ensure that the reaction rate did not depend on agitation speed. During the reaction, the liquid samples were withdrawn by the syringe from the reactor with an appropriate septum and analyzed by gas chromatography with FID detector using Perkin Elmer Clarus 500. Concentrations of PhAc, styrene and ethylbenzene in the reaction mixtures were determined by comparison with calibration curves using *n*-decane as a standard.

## 4. Conclusions

In this work, poly((hydromethylsiloxane) (PHMS) was cross-linked with 1,3,5,7-tetramethyl-1,3,5,7-tetravinylcyclotetrasiloxane (D_4_^Vi^) in high internal phase water-in-oil emulsions to form macroporous materials, called polyHIPEs. The studies conducted lead to the following conclusions:

1. PHMS-based polyHIPEs at 520 °C in Ar atmosphere transform into hybrid inorganic-organic materials (ceramers) that contain predominantly macropores and micropores, but also a lower amount of mesopores.
2. Pyrolysis affects the sizes and size distributions of macropores present in the initial polyHIPEs.
3. Total pore volume and specific surface area (SSA) of ceramers, related to the presence of micro- and mesopores, depend on the amount of the cross-linker applied in the synthesis of a polyHIPE. They lower as the amount of the cross-linker during polyHIPE preparation grows.
4. Impregnation of ceramers with palladium(II) acetate solution in toluene followed by heating at 350 °C in air leads to PdO/ceramer composites. Reduction by hydrogen of PdO present in the materials to metallic Pd is easier in the case of the composites prepared using ceramer matrices of lower porosity.
5. PdO/ceramer composites, after reduction with H_2_, are active and selective catalysts in the hydrogenation of phenylacetylene to styrene under mild conditions. Ceramers of lower total pore volume and SSA should be preferred for use as supports for the catalysts as they ensure higher catalytic activity.

To summarize, we conclude that macro-/micro-/mesoporous hybrid ceramics can be easily obtained in the process of pyrolysis of polysiloxane polyHIPEs. Their total pore volume and SSA can be controlled by changing the cross-linking agent/polymer ratio in the polyHIPE synthesis. These materials can find applications in the area of catalysis and in other areas where the presence of pores of various sizes is required.

## Figures and Tables

**Table 1:** Mass losses of P1–P3 polyHIPEs at selected temperatures determined by TG studies.

Sample	Mass Loss [wt.%]						
	450 °C	520 °C	650 °C	750 °C	1000 °C	1200 °C	1500 °C
P1	11.4	15.4	21.2	21.7	22.6	23.5	25.6
P2	8.2	12.0	16.2	17.7	19.9	21.0	23.0
P3	13.1	17.2	20.0	22.4	23.0	23.2	24.0

**Table 2:** Results of SEM image analysis of the studied materials conducted using ImageJ 1.53k software.

Sample	Voids					Windows				
	Diameter [μm]				Low Diameter (<4 μm) Fraction [%]	Diameter [μm]				Low Diameter (<0.6 μm) Fraction [%]
	Minimum	Maximum	Mean	Median		Minimum	Maximum	Mean	Median	
P1	2.03	9.89	9	4.70	30.5	0.27	1.81	0.71	0.66	43.3
P2	1.16	8.82	3.68	3.44	64.6	0.08	1.90	0.74	0.68	36.4
P3	1.40	11.20	4.03	3.76	56.1	0.23	2.36	0.74	0.67	40.9
C1	1.28	9.60	3.69	3.41	68.4	0.18	2.79	0.85	0.69	38.3
C2	1.08	9.37	3.08	2.77	80.2	0.13	1.79	0.52	0.48	71.6
C3	2.54	9.03	5.20	4.95	18.6	0.36	2.17	0.79	0.75	29.0

**Table 3:** Results of analysis of N_2_ adsorption data.

Sample	Pore Volume [cm^3^/g]			Micro-/ Mesopore Volume Ratio	S_BET_ [m^2^/g] (BET Constant, C)	S_ext_ [m^2^/g]
	Total	Micropores	Mesopores			
C1	0.143	0.089	0.054	1.6	344 (C = 2340)	23
C2	0.041	0.022	0.019	1.2	113 (C = 430)	18
C3	0.036	0.026	0.010	2.6	92 (C = 850)	5

**Table 4:** Initial rate, phenylacetylene conversion at maximum styrene yield, styrene maximum yield and selectivity (S_max_) in phenylacetylene hydrogenation process catalyzed by the C1_Pd-C3_Pd-derived materials.

Sample	Initial Rate of Hydrogenation [mol/min⋅g Pd]	Phenylacetylene Conversion [%]	Styrene Maximum Yield [%]	S_max_ [%]
C1_Pd	0.06	92	81	88.0
C2_Pd	0.16	85	76	89.4
C3_Pd	0.14	90	78	86.7

## Data Availability

The original contributions presented in the study are included in the article, further inquiries can be directed to the corresponding author.
